# Targeting Head and Neck Cancer by Vaccination

**DOI:** 10.3389/fimmu.2018.00830

**Published:** 2018-04-23

**Authors:** Chuan Wang, James Dickie, Ruhcha V. Sutavani, Catherine Pointer, Gareth J. Thomas, Natalia Savelyeva

**Affiliations:** Cancer Sciences Unit, Faculty of Medicine, University of Southampton, Southampton, United Kingdom

**Keywords:** head and neck cancer, human papillomavirus, human papillomavirus independent, cancer antigens, cancer vaccines

## Abstract

Head and neck cancer (HNC) is a heterogeneous group of squamous cell cancers that affect the oral cavity, pharynx, and larynx. Worldwide, it is the sixth most common cancer but in parts of Southern and South-East Asia, HNC is one of the most common cancers. A significant proportion of HNC is driven by human papillomavirus (HPV) infection, whereas HPV-independent HNC is associated with alcohol, smoking, and smokeless tobacco consumption. Here, we review the past and present experience of targeting HNC with vaccination focusing on HPV-derived antigens as well as non-viral antigens for HPV-negative HNC. Novel therapeutic approaches for HNC will focus not only on effective vaccine platforms but will also target the stroma-rich immunosuppressive microenvironment found in those tumours.

## Introduction

Head and neck cancer (HNC) is a heterogeneous group of squamous cell cancers that affect the oral cavity, pharynx, and larynx. Overall, it is the sixth most common cancer worldwide with an annual estimated incidence of 550,000 cases and around 300,000 deaths (1–3). In parts of Southern and South-East Asia, HNC is one of the most common cancers, and the actual incidence in these developing countries is probably underestimated (1, 4, 5). While the aetiology of HNC is usually associated with smoking and alcohol, a significant subset of oropharyngeal cancers is driven by human papillomavirus (HPV) infection, and these cancers account, at least in part, for the significant increase in HNC in recent years (3, 6, 7).

Combinations of surgery, chemotherapy, and radiotherapy form the standard current first-line treatment regimens for HNC. But despite improvements, these are associated with significant morbidity and a relatively static 5-year survival rate of around 40–50% (1). HPV-positive HNCs have a better prognosis than HPV-negative HNC. Recent clinical trials have demonstrated a clear survival advantage in advanced head and neck squamous cell carcinoma patients treated with immune checkpoint blockade [for review, see Ref. (8)]. In a recent KEYNOTE 012 trial, treating HNC patients with anti-PD1 produced an overall response rate of 24.8% (9). Most patients (around 80%), however, do not respond to checkpoint inhibitor monotherapy aimed to boost pre-existing anti-tumour immune responses. The focus is now on induction of anti-tumour immune responses using cancer vaccines.

## HPV-Positive Versus HPV-Negative HNC

The incidence of HNC has risen dramatically since the later 1970s and this has been linked to HPV infection. Around 25–50% of HNCs are HPV-driven with a higher percentage in developed countries (10–14). This percentage is expected to increase in coming years due to many patient cohorts being infected before prophylactic vaccination against HPV started. HPV-positive HNC predominantly tend to be restricted to the oropharynx, conversely most oropharyngeal cancers are reportedly HPV-positive (11).

Human papillomavirus is an asymptomatic, sexually transmitted DNA virus that infects squamous epithelium *via* micro abrasions which expose the deeper basal epithelial cells (15). Most of HPV-positive HNC (90%) are driven by high-risk HPV16 in contrast to 70% of cervical cancers that are linked to either HPV16 or 18 (11, 13, 16, 17). Other high-risk types involved include HPV 18, 31, and 33. The HPV genome encodes eight genes which are expressed early (E1, E2, E4, E5, E6, and E7) or late (L1 and L2) in the virus life cycle. E6 and E7 are the first viral proteins expressed following infection (15). They inhibit tumour suppressors p53 and pRb, respectively, resulting in uncontrolled host DNA synthesis and cell division; the first step towards malignant transformation (16). E2 protein differentially regulates E6/E7 expression through control of their transcription (18, 19). E5 is known to play an anti-apoptotic role and is thought to contribute to the early stages of oncogenesis (20, 21) by cooperating with E6 and E7 to immortalize cells (22). E5 is not necessary for the maintenance of the transformed phenotype and is often lost. L1 and L2 are structural proteins and form the viral capsid required for infectious viral particles (23).

For HPV-negative HNC incidence, habitual and cultural factors play a major role. In high-income countries, smoking and alcohol [70 and 30%, respectively, or 80% combined (24)] contribute, while in developing countries of Southern and South-Central Asia, HNC and in particular oral squamous cell carcinoma (OSCC), are primarily linked to smokeless tobacco and paan (1). Chewing of paan or betel quid has been strongly attributed to both OSCC and oral premalignancy (25). Besides tobacco, areca nut included in betel quid is also a known carcinogen and the mixture of tobacco, areca nut, and slaked lime forms a potent carcinogenic combination.

HPV-negative HNC also differs from HPV-positive genetically, and common genetic alterations which lead to inactivation of cell-cycle suppressors p53 and p16 and amplification of CCND1 (cyclin D) have been found in the HPV-negative HNC subset. Further alterations in the genes associated with smoking such as those involved in oxidative stress CUL3, KEAP1, and NFE2L2 are also associated with the HPV-negative subset (26–28).

## Prophylactic Vaccination Against HPV

**Figure 1:**
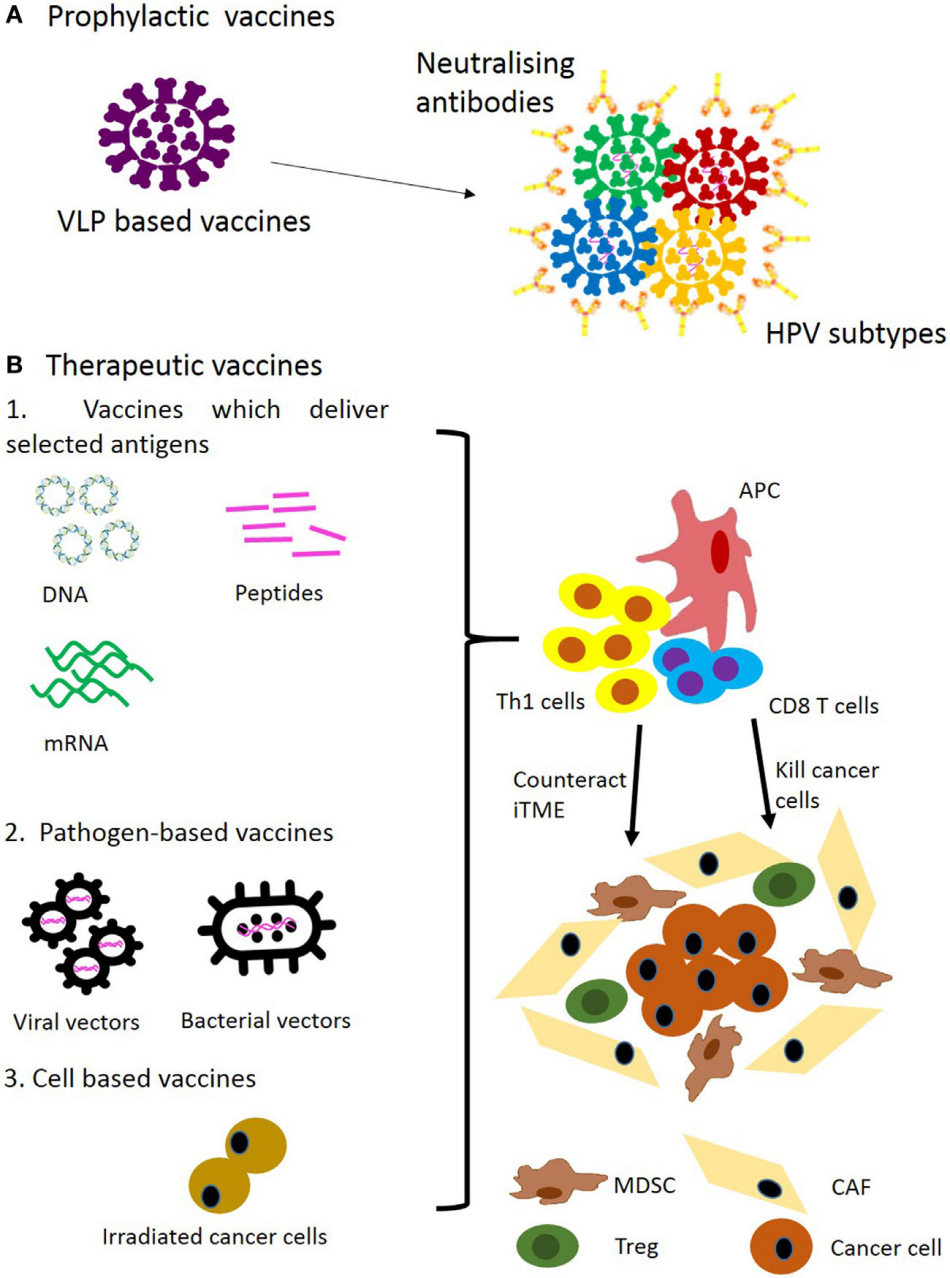
Approaches for prophylactic **(A)** and therapeutic **(B)** vaccination for head and neck cancer. Abbreviations: VLP, virus-like particle; iTME, immunosuppressive tumour microenvironment; MDSC, myeloid-derived suppressor cell; Treg, regulatory T cell; CAF, cancer-associated fibroblast.

Several prophylactic vaccines including Cervarix, Gardasil^®^ and more recently Gardasil^®^9 have been approved by the FDA to protect from HPV infection as well as HPV-associated diseases such as genital warts and cancer (Figure 1A) (29–32). The prophylactic effect specifically on HNC is assumed without relevant epidemiological studies available at present.

These vaccines are based on virus-like particles (VLPs) consisting of different HPV capsid proteins L1 (33). For example, Gardasil consists of VLP derived from genital warts-inducing HPV6 and 11, and oncogenic strains of HPV16 and 18. One VLP is made of one type L1 molecule. When L1 is expressed using recombinant protein expression systems it self-assembles into VLPs *in vitro* (34–36). Superior properties of VLPs in induction of antibody are largely accounted for by their multimeric structure, and their ability to stimulate naive B cells has been demonstrated (37). Prophylactic HPV vaccines target the viral infection itself by inducing neutralising antibody and are effective in preventing HPV-induced malignancies but are not effective in treating them (38). The vaccine target L1 is not expressed during the oncogenic process. Hence, antigens expressed in the tumour have to be targeted by therapeutic vaccination.

## Therapeutic Cancer Vaccines

### Target Antigens

For HPV-positive cancers, the expressed viral antigens are available. For HPV-negative cancers, other antigens have to be considered. Cancer antigens can be broadly classed into two categories: tumour-specific antigens (TSAs) and tumour-associated antigens (TAAs). Here, we describe TSAs as proteins only expressed in cancer cells and mutated self-proteins (neoepitopes), and TAAs as unmutated self-proteins such as glycosylated proteins MUC1 and CEA or cancer testis antigens (CTAs) (39). TSAs often generate strong immune responses, but are comparatively less available than TAAs. TAAs are generally well conserved in populations, but tend to generate a weaker immune response (40, 41).

To cover antigens which have been targeted to date, current clinical trials for HNC were queried at the NIH ClinicalTrials.gov database utilising “Head and Neck Cancer” or “Oropharyngeal Cancer” or “Oral Cancer” as the disease, and “Vaccine” as the search string, yielding 55 studies. Terminated, withdrawn, suspended trials as well as trials with unknown recruitment statuses were excluded and the results are summarised in Table 1. The WHO International Clinical Trials Registry Platform was also queried with the same search strings yielding two additional trials not included on the NIH ClinicalTrials.gov database, but registered on the Japanese UMIN-Clinical Trials Registry (UMIN000008379, UMIN000000976).

**Table 1:** Target cancer antigens in head and neck cancer.

Vaccine	Target antigens	Type	Phase	Identifier	Relevant references
**Pre-active/active clinical trials**					

ADXS11-001	Human papillomavirus (HPV)16-E6/E7	Viral Ag	Phase II	NCT02002182	(136)
			Phase I/II	NCT02291055	N/A
DPX-E7	HPV16-E7	Viral Ag	Phase I/II	NCT02865135	N/A
MEDI-0457 (INO-3112)	HPV16/18-E6/E7	Viral Ag	Phase I/II	NCT03162224	N/A
TG4001	HPV16-E6/E7	Viral Ag	Phase I/II	NCT03260023	N/A
ISA101/101b	HPV16-E6/E7	Viral Ag	Phase II	NCT02426892	N/A
			Phase II	NCT03258008	N/A
ISA201 (Hespecta)	HPV16-E6/E7	Viral Ag	Phase I	NCT02821494	(121)
HARE-40	HPV16-E6/E7	Viral Ag	Phase I/II	NCT03418480	N/A
Trojan	MAGE-A3 and HPV16-E7	Viral Ag and tumour-associated antigen (TAA)	Phase I	NCT00257738	(81)
MUC1 vaccine	MUC1	TAA	Phase I/II	NCT02544880	N/A
NANT	MUC1/CEA/HER2/Brachyury/Ras	TAA	Phase I/II	NCT03169764	N/A
MVX-ONCO-1	Allogeneic tumour-irradiated	Cellular	Phase II	NCT02999646	N/A
AlloVax	Allogeneic tumour-chaperone-rich cell lysate	Cellular	Phase I/II	NCT01998542	N/A
			Phase II	NCT02624999	N/A

**Completed clinical trials**					

Peptide pulsed dendritic cell	P53	TAA	Phase I	NCT00404339	(69)
Ras vaccine	Ras	TAA	Phase II	NCT00019331	N/A
MEDI-0457 (INO-3112)	HPV16/18-E6/E7	Viral Ag	Phase I/II	NCT02163057	(111)
P16 vaccine	P16	TAA	Phase I/II	NCT01462838	(120)
			Phase I	NCT02526316	N/A
GI-6207	CEA	TAA	Phase I	NCT00924092	N/A
EBV vaccine	EBV	Viral Ag	Phase I	NCT01147991	(58)
TRICOM-CEA(6D)	CEA	TAA	Phase I	NCT00027534	N/A
Peptide with IFA	CDCA1, LY6K, and IMP3	TAA	Phase I/II	UMIN000008379	(87)
Survivin-2B vaccine	Survivin-2B	TAA	Phase I	UMIN000000976	(70)

#### Viral Antigens

Viral proteins are considered to be good targets since they are foreign, and hence, the available T-cell repertoire has not been subjected to central tolerance. Most vaccination strategies for HNC target the HPV-positive subset where HPV antigens can be used. HPV E6 and E7 play a critical role in carcinogenesis of HNCs, similar to ano-genital cancers. During malignant transformation when HPV frequently integrates into the host genome, E6 and E7 are thought to be the only proteins expressed and hence have been targeted by many types of vaccines. Vaccines against these antigens have demonstrated efficacy in HPV-induced cervical dysplasia (42–46) and are currently in clinical trials for both cervical cancer (e.g., NCT02128126) and HNC (Table 1).

Other potential targets are E2 and E5. E2 has been successfully targeted in ano-genital intraepithelial lesions (47). In HNC, E2 is not always lost, and can be retained in episomal HPV DNA (48) (and unpublished data from our lab). A number of vaccines targeting E5 are in preclinical development (49–52). No clinical data on targeting E2 and E5 in HNC are currently available.

HPV-negative HNC lacks the immunogenic HPV viral proteins of HPV-associated HNC and appears less responsive to current treatments (53). A viral target which may be present in HPV-negative cases is EBV, which is strongly associated with nasopharyngeal cancer (54), though only around 6–21% of HNC cases express EBV RNA (55, 56). Vaccines targeting EBV are currently in phase I clinical trials (Table 1; NCT01147991), and appear safe and well tolerated, inducing only grade I/II adverse events, while reporting increased circulating CD4 cells and antigen-specific T cell responses (57, 58).

#### Neoepitopes

Despite earlier reports that HPV-negative HNC had a greater mutation rate than HPV-positive (59), more recent reports have found no significant difference to the mutation rate as a result of HPV status (26, 27). However, these reports do find significant differences in the mutational spectrum based on HPV status, which influence vaccine-targetable mutations. Targeting p53 and RAS is more likely to benefit HPV-negative cases, as these proteins are mutated in HPV-negative cases, but degraded in HPV-positive cases (60). Early trials targeting mutated p53 or RAS have been completed (61). The RAS phase II trial was completed in 2007, but no results have been reported to date (Table 1; NCT00019331).

Conventional mutation targeting in cancer therapy focuses upon driver mutations, but in the last decade has arisen a view that other mutations may be relevant and make for potential vaccine targets (62, 63). Advances in immunotherapeutics and bioinformatics in recent years have increased the practicality of targeting these neoantigens *via* vaccination. However, as each case exhibits its own unique mutanome (64), each vaccine must be created for the specific individual, making it expensive and time consuming. Recent studies reported around 100 days are required for production and analytical testing (NCT02035956, NCT01970358) (65, 66). Despite the current great expense of both time and money, early results suggest great efficacy from targeting neoantigens (65, 66).

#### Tumour-Associated Antigens

Well-characterised antigens including MUC1 and CEA have demonstrated immunogenicity in patients and their potential is being translated into clinical efficacy (67, 68). In HNC, phase I/II trials targeting MUC1 are on-going (Table 1; NCT02544880), while trials targeting CEA (Table 1; NCT00924092, NCT00027534) have been completed but have yet to report results. The p53 phase I trial targeting wt p53 T-cell epitopes completed with modest improvement to 2-year disease-free survival (DFS) [Table 1; NCT00404339 (69)], but has yet to progress to a phase II trial. A phase I trial targeting Survivin-2B has been performed in oral cancer (Table 1; UMIN000000976), but demonstrated low efficacy (70). The NANT vaccine (Table 1; NCT03169764) is a novel combination immunotherapy combining metronomic chemo-radiotherapy with vaccines targeting well-established and molecularly confirmed TAAs, and off-the-shelf NK cell therapy. This experimental therapy is part of the Cancer Breakthroughs 2020 global initiative.

Cancer testis antigens are a class of TAAs that make for promising vaccine targets. While there is evidence of central tolerance for CTAs (71), CTA expression in the periphery is normally restricted to healthy male germ cells, immune privileged cells lacking MHC I (72). Thus, CTAs are only presented to the immune system in the periphery by cancer cells (73), and thus frequently demonstrate immunogenicity (74–76).

Using either SEREX or TIL-derived T cells, many CTAs have been described over the past decades, most prominently the MAGE family, BAGE family, SSX family, PRAME, and NY-ESO1 (77–79). A recent analysis of HNC selected several potential CTA targets for further preclinical study, although they did not differentiate between HPV-associated and HPV-negative cases (80). A phase I clinical trial targeting MAGE-A3 and HPV antigens in HNC is on-going (Table 1; NCT00257738) (81).

Novel CTAs including LY6K, CDCA1, and IMP3 have been identified through genome wide microarray analysis of various cancer tissues (82–84). A multivalent vaccine targeting HLA-A24 restricted LY6K, CDCA1, and IMP3-derived peptides recently was tested in phase I and phase II clinical trials in oesophageal cancer (85, 86), and significantly improved DFS in HLA-A24 cases was reported. This success encouraged targeting of these antigens in HNC (Table 1; UMIN000008379) (87), which increased overall survival (OS) when administered to HLA-A24 patients with advanced refractory HNC (HPV-negative with the exception of one patient) and correlated with peptide specific CTL responses.

### Immune Mechanisms for Cancer Attack

Most target antigens in HNC including HPV antigens, neoepitopes as well as the majority of TAA are intracellular antigens. Intracellular antigens are generally presented as 8-11-mer peptides bound to MHC I on cancer cells and these are targeted by CD8 cytotoxic T cells (CTLs). CTLs are powerful effector cells which directly kill target cells *via* a variety of mechanisms including perforin/granzyme and Fas-mediated attack. For induction of long-lasting CTLs, CD4 T helper (Th)1 cells must also be co-induced (88). These provide T-cell help *via* dendritic cells (DC)-licensing by binding to 12-15mer or longer peptides presented in the context of MHC II on DCs which leads to DC activation (89, 90). These Th1 subsets do not have to be specific for the target cancer antigen. Approaches for recruiting Th1 cells specific for foreign antigens have been extensively explored in the development of cancer vaccines (91–94). The advantage of this approach is that these Th cells escape tolerogenic mechanisms and are therefore available to provide help to CTLs specific for cancer-derived antigenic peptides [for review, see Ref. (95)]. Tetanus-derived Dom helper sequence has been used to recruit CD4 T cell for induction of CD8^+^ CTLs using DNA vaccine (96). A recent phase II clinical data demonstrated this approach led to induction of CD8^+^ T-cells response to CEA detected post-vaccine with indication of clinical benefits (67).

For effective cancer attack, Th1 cells that play not only a helper role to CTLs but a more direct role in anti-tumour immunity are important (97–99). Those specific for the target antigen Th1 cell subsets may be involved in recruitment of tumouricidal macrophages or reprogramming of the tumour microenvironment (100, 101). In HPV-associated cancer, targeting co-induction of broad CD4 and CD8 T-cell responses correlated with vaccine efficacy (44). This is in keeping with the data obtained in other solid tumours (65, 66).

## Vaccine Platforms

Delivering antigenic epitopes in an immunogenic context which leads to the induction of a durable T-cell response is the goal. The available vaccine platforms are illustrated in Figure 1B. DNA and RNA vaccines encoding selected tumour antigens or synthetic long peptides (SLPs) vaccines co-delivering CD4 and CD8 epitopes have recently been highlighted as optimal cancer vaccine modalities (97). These focus on delivery of selected target antigens without co-delivering the backbone-encoded antigens as in the case of pathogen-derived bacterial or viral vectors. The latter often generate strong pathogen-derived CD8 epitopes and can focus the immune response on the vector itself (96). Nevertheless, several viral and bacterial vaccines have demonstrated induction of CD8 responses against dysplastic disease and cancer together with clinical efficacy (47, 68).

### DNA Vaccines

DNA vaccines represent a simple approach of directly injecting a plasmid DNA encoding one or more antigens driven by a eukaryotic promoter. Not only is the antigen made directly in the body but the DNA backbone also acts as an immunological adjuvant (102). Multiple innate sensors for plasmid DNA have been identified including endosomal toll-like receptor (TLR) 9 as well as several cytosolic sensors DAI, AIM2, cGAS-STING, and others (103). Flexibility, simplicity of preparation, stability, and safety are the advantages. However, low immunogenicity in patients has been highlighted in early clinical trials (104). The situation improved upon combination of DNA vaccine injection and *in vivo* electroporation [EP, for review, see Ref. (105)]. *In vivo* EP increased cellular DNA uptake leading to generation of more antigen available for immunisation and potentially made DNA more visible to the cytosolic innate sensors. This led to significant increase in immunogenicity. Combination of DNA and EP induced durable antibody and T-cell responses in cancer patients (42, 67, 104, 106). Other methods of DNA delivery including liposomes, tattooing and cationic polymers have also been also investigated (107, 108).

For targeting of HPV, oncogenes E6 and E7 by DNA vaccines modifications have been made to their sequences to prevent E6 and E7 binding to p53 and pRb, respectively (42, 109, 110). Most HPV-targeting DNA vaccines so far have been trialled in the setting of cervical intraepithelial neoplasia (CIN). In the recent phase IIb clinical trial, VGX-3100 DNA vaccine encoding E6 and E7 in combination with DNA vaccine encoding IL-2 administered i.m. with EP has shown promising clinical results in women with HPV16- and 18-associated CIN2/3. Robust T-cell responses were induced and regression of premalignant lesions was demonstrated in 50% of vaccinated women (42). VGX-3100 was subsequently moved to the HNC setting where it was also delivered with EP plus DNA vaccine encoding IL-12 (the combined treatment defined as INO-3112; Table 1; NCT02163057). Initial results from the study were very promising. HPV E6/E7-specific antibody was successfully generated in four of the five HNC patients analyzed. Increased HPV-specific cellular responses were observed in nine out of 10 evaluable patients by ELISPOT. Seven of eight evaluable patients had HPV-specific granzyme/perforin positive CD8 T cells by flow cytometry (111). A phase I/II trial to assess the vaccine (now called MEDI-0457, MedImmune) safety and anti-tumour efficacy in combination with PD-L1-blocking mAb Durvalumab is now recruiting HPV-positive HNC patients (Table 1; NCT03162224).

DNA vaccine targeting HPV16 E7, pNGVL4a-CRT/E7 (detox), based on E7-calreticulin (CRT) fusion demonstrated the ability to enhance MHC I presentation and exhibited an anti-angiogenic effect (112). In a preclinical study, E7-specific antibody and T-cell responses were generated with protection from the TC-1 tumour challenge (113). A pilot clinical study using pNGVL4a-CRT/E7 (detox) for the treatment of patients with HPV16-associated CIN2/3 was recently conducted (114). EP was not used in this study but one arm investigated the particle-mediated epidermal delivery using a needle free ND10 delivery system. The results demonstrated mild but manageable toxicity predominantly localised to the injection site but only a small increase in systemic T-cell responses was observed with no increase in regression above the control. A phase I trial assessing safety and feasibility of this DNA vaccine in combination with cyclophosphamide in HPV16-associated HNC patients has been terminated (Table 1).

Immunogenicity and efficacy of a novel linear closed end DNA vaccine, doggybone (db) DNA (dbDNA™, Touchlight Genetics), have recently been demonstrated in the HPV E6 and E7 tumour model (115). dbDNA™ vaccine was developed using a bacteria-free manufacturing platform which relies on bacteriophage Phi29 polymerase for amplification. Minimal purification is required and safety is improved because of exclusion of antibiotic-resistant genes irrelevant for this platform. dbDNA™ vaccine operated through STING-mediating pathways but was independent of TLR9 recognition. Importantly, HPV16 E6 and E7 dbDNA™ vaccine and conventional plasmid DNA delivered with EP generated similar levels of CD4 and CD8 T cells as well as antibody. dbDNA™ was also able to suppress established TC-1 tumours similar to plasmid DNA. This novel DNA vaccine represents a promising alternative to a plasmid DNA vaccine for targeting of HPV E6 and E7 antigens.

### mRNA Vaccines

mRNA vaccines are becoming increasingly attractive in recent years. They can accumulate at high concentration in the cytoplasm ensuring high antigen expression. mRNA is a natural ligand for TLR3, TLR7/8, and several cytosolic sensors (i.e., RIG-I, MDA5), which induce innate immune response to enhance vaccine efficacy [for review, see Ref. (116)]. For *in vivo* delivery, mRNA is complexed with a lipid carrier that protects from degradation as well as targets DCs (117). The efficacy of a first-in-human personalised mRNA vaccine targeting patients’ mutanome in melanoma patients has been reported recently (65). T-cell responses against multiple mutanome-derived neoepitopes were induced in all patients. Four out of five patients with progressing metastasis at the start of vaccination demonstrated either a partial or a complete clinical response after vaccination. Interestingly, one patient benefited from vaccination followed by anti-PD1 mAb. A phase I/II clinical trial using an mRNA vaccine targeting HPV16 E6 and E7 has recently started recruiting at our institution (University of Southampton, led by Prof C. Ottensmeier and Dr E. King; NCT03418480). The vaccine will be given to patients with HPV-positive HNC intradermally either alone or in combination with anti-CD40 costimulatory antibody.

### Peptide Vaccines

Peptide-based vaccines are safe and easy to produce, but they are also expensive and poorly immunogenic by themselves. They are often CD8^+^ epitopes predicted for a particular HLA allele or include long single or overlapping peptides which often contain both CD8 and CD4 epitopes. The latter approach circumvents HLA restriction issues. Unlike DNA and RNA vaccines peptides do not carry “inbuilt” adjuvants. The efforts to enhance their immunogenicity have focused on combining with appropriate adjuvants. MF59^®^, emulsion of squalene oil approved in both Europe and USA, has been used in earlier trials (97). A number of clinical trials have used SLPs targeting HPV antigens combined with oil-in-water adjuvants for treatment of CIN and vulvar neoplasia which resulted in regression of premalignant lesions (43–46). However, this approach has not been successful when tested in recurrent cervical cancer with low T-cell responses and no survival benefit (118). This failure highlighted the need to use more potent adjuvants and combinational therapeutic approaches to overcome the tumour microenvironment (119).

Several peptide-based vaccines against HPV-associated HNCs are now in clinical trials. A phase I/II trial to assess safety and efficacy of a short peptide-based vaccine targeting HPV16 E7 (11–19), in combination with low dose of cyclophosphamide intending to deplete regulatory T cells, has started recently in HLA-A2 patients with incurable HPV16-associated oropharyngeal, cervical, and anal cancer (Table 1; NCT02865135). In another clinical trial, patients with advanced HPV-associated cancers were vaccinated weekly with a SLP derived from p16 (27, 37–62), the tumour suppressor induced as a result of HPV-linked transformation, after the completion of a standard treatment. The vaccine containing both CD8 and CD4 epitopes was emulsified with Montanide™ ISA-51 VG (oil-in-water adjuvant, SEPPIC). Both cellular and humoral responses to the peptide were induced with no unexpected serious adverse reactions. Out of 14 evaluated patients, nine had stable disease as their best overall response and five patients developed progressive disease [Table 1; NCT01462838 (120)]. A subsequent on-going trial using the same vaccine has been evaluating different routes of vaccination, i.e., subcutaneous and intradermal (Table 1; NCT02526316). Two phase II clinical trials using 13 HPV16 E6/E7 overlapping SLPs in combination with anti-PD-1 antibody (Nivolumab) or in combination with anti-CD137 immuno-stimulatory antibody (Utomilumab) have been initiated to treat patients with HPV16-associated HNC as well as other HPV-associated malignancies (ISA101/ISA101b; Table 1; NCT02426892 and NCT03258008) (119). A phase I trial using two HPV16 E6 SLPs (ISA201) together with TLR1/2 agonist adjuvant Amplivant^®^ (ISA pharmaceutical), for HPV16-positive tumours and premalignant lesions has also been initiated [Table 1; NCT02821494 (121)].

A multivalent vaccine targeting HLA-A24 restricted short peptides from three CTAs (LY6K, CDCA1, and IMP3) in combination with IFA injected subcutaneously has recently cleared phase II clinical trials in HNC patients in Japan [Table 1; UMIN000008379 (87)]. The vaccine increased OS when administered to HLA-A24 patients, which was correlated to peptide specific CTL responses. Interestingly, those patients that demonstrated response to all three peptides had extended OS versus those who responded to one or two peptides only.

### Viral and Bacterial Vector-Based Vaccines

Viral vector-based vaccines employ attenuated viruses that deliver antigen of interest in the infected cells. Alphaviruses, adenoviruses, and vaccinia viruses are the examples that have been explored to deliver HPV-associated antigens.

Replication-deficient alphaviruses including Semliki Forest virus (SFV) and Venezuelan equine encephalitis virus (VEE) have been demonstrated to be safe [for review, see Ref. (122)]. These RNA viruses preferentially infect APCs and are able to efficiently activate the adaptive immune system [for review, see Ref. (123)]. SFV- and VEE-based vaccines against HPV16 E6/E7 have demonstrated the ability to induce specific CTLs that can kill HPV16 E6/E7-expressing tumour cells *in vitro* and clear tumours in mouse models (124–126), including in the HLA-A*0201 transgenic mice (127, 128).

The first clinical trial of a recombinant vaccinia virus targeting HPV was conducted more than 20 years ago. The TA-HPV vaccine was based on a live vaccinia virus and was engineered to express E6 and E7 proteins from HPV16 and 18. Two patients with advanced cervical cancer remained tumour free 15 and 21 months after vaccination, in one of them an HPV-specific T-cell response was also induced (129). Two more clinical trials using the same vaccine to treat HPV-associated advanced cervical cancer and vulval/vaginal intraepithelial neoplasia had been reported with partly successful results, but also some side effects manifesting as erythema and swelling followed by ulceration with scab formation at the site of vaccination (130, 131). Safety concerns related to the use of live vaccinia virus prompted the development of vaccines based on the attenuated virus, i.e., modified vaccinia virus Ankara (MVA) (132, 133). MVA also preferentially infects APCs (134) and through recognition of its viral DNA by TLR9 and cytosolic DNA sensors is able to activate APCs leading to effective activation of T-cell immunity. The safety as a result of restricted replication has been demonstrated in many clinical trials (132).

Efficacy of an MVA-based vaccine encoding modified HPV16 E6 and E7 together with IL-2 TG4001 (Transgene) was demonstrated in patients with HPV16-related CIN2/3. In 7 out of 10 patients who were evaluated as clinical responders, cytological and colposcopic regression together with HPV16 mRNA clearance was observed (135). A phase I/II clinical trial assessing TG4001 in HPV16-associated HNC patients has recently started recruiting (Table 1; NCT03260023).

A well-characterized attenuated facultative intracellular bacterium *Listeria monocytogenes* (Lm) incorporating a non-toxic version of the listerionlysin O (LLO) as adjuvant has been utilised to target HPV16 E7 oncoprotein (Lm-LLO-E7 also known as AXAL, ADXS11-001) [(136); for review, see Ref. (137)]. Several clinical trials using this vaccine in cervical and anal cancers are on-going, including one which progressed to phase III last year in women with high-risk locally advanced cervical cancer (NCT02853604). With regard to HNC, one phase I clinical trial in oropharyngeal cancer was terminated in 2016 as one patient suffered a dose limitation toxicity (NCT01598792). Two more phase II trials using the vaccine in combination with anti-PD-L1 mAb (Table 1; NCT02291055) or surgery (Table 1; NCT02002182) are still active and recruiting. The latter trial has recently reported specific T-cell responses in the blood and increased T-cell infiltration in the tumour in five out of eight and four out of eight patients, respectively (138).

### Cellular Vaccines

Using autologous tumour cells as vaccines ensures that patients are vaccinated with cells containing the same tumour antigens that their tumour expresses saving time and effort needed to identify TSAs. Irradiated cells are used but undesired immune responses are still a potential safety concern. To enhance immunogenicity cells genetically modified to express costimulatory molecules, TLR ligands or cytokines have been utilised (139). Phase I/II trials are underway for personalised HNC vaccines Allovax and MVX-ONCO-1, utilising allogeneic tumour cells as a source of antigen (Table 1; NCT02999646, NCT01998542, NCT02624999). MVX-ONCO-1 contains irradiated autologous tumours cells expressing GM-CSF combined with encapsulated cellular technology that allows continuous supply of GM-CSF (140). It has demonstrated reasonable safety in a phase I trial for solid tumours, with no systemic serious adverse events (NCT02193503) (141). AlloVax™ is Charperone Rich cell lysate combined with AlloStim™ cells which are allogeneic Th1 effector cells [Allostim, Immunovative Therapies Ltd. (142)].

## The Role of the Immunosuppressive Tumour Microenvironment: Cancer-Associated Fibroblasts (CAFs)

Several immunosuppressive immune subsets have been found in HNC including tumour-associated macrophages, myeloid-derived suppressor cells, and regulatory B and T cells. The role of these have been reviewed elsewhere (143, 144), but it is clear that vaccination must induce the correct immune milieu for therapy to be effective. Similarly, we have identified several features of the tumour microenvironment, including tumour cell glycolysis/hypoxia and a CAF-rich stroma that are associated with “immune cold” HNC (145, 146). It has been suggested that CAFs suppress T-cell infiltration into cancers, and also through secretion and activation of TGF-β, modulate multiple types of immune cells towards a more suppressive phenotype, including tolerization of CD4 T cells and promotion of a regulatory T cell phenotype. Tumours with this type of stromal response may not be effectively targeted by vaccination; it is possible, however, that such evasion mechanisms could be targeted as part of a vaccination strategy; for example, we have shown recently that CAFs can be specifically targeted by inhibiting the NADPH oxidase, NOX4 (146).

## Concluding Remarks

Incidence of both HPV-positive and -negative HNC is on the increase. The trend is unlikely to change at least in the near future, particular for HPV-independent HNC, especially OSCC, where the habitual and cultural causes are unlikely to disappear.

Human papillomavirus targets E6 and E7 are considered to be less challenging because of their foreign nature. Their targeting by DNA, peptides, and other vaccines has already demonstrated clinical efficacy in HPV-driven dysplasia. These vaccines are now in clinical trials for HPV-driven cancers including HNC. On the contrary, HPV-independent HNC has received less attention largely because such targets have not been available. A number of interesting antigenic targets has started coming through; these include personalised mutanome-derived neoepitopes but also novel TAAs (87). The mutanome-based approach has started demonstrating clinical efficacy but it is unlikely to have an impact on the disease in the developing parts of the world. Therefore, the focus is on TAAs.

More affordable vaccine modalities such as DNA vaccines combined with a simple *in vivo* delivery are promising. However, these will still need to be developed within a strategy that overcomes a suppressive tumour microenvironment and further work is required to develop these therapeutic approaches.

## Author Contributions

CW, JD, RS, and NS wrote sections on target cancer antigens, immune mechanisms, and vaccine platforms. CP and GT wrote sections on HNC and tumour microenvironment. All authors contributed to discussion of the manuscript.

## Conflict of Interest Statement

The authors declare that the research was conducted in the absence of any commercial or financial relationships that could be construed as a potential conflict of interest.
